# Does a Flatter General Gradient of Visual Attention Explain Peripheral Advantages and Central Deficits in Deaf Adults?

**DOI:** 10.3389/fpsyg.2017.00713

**Published:** 2017-05-16

**Authors:** Vincent J. Samar, Lauren Berger

**Affiliations:** ^1^NTID Department of Liberal Studies, Rochester Institute of Technology, RochesterNY, USA; ^2^PhD Program in Educational Neuroscience, Gallaudet University, WashingtonDC, USA

**Keywords:** deafness, attention, cross-modal plasticity, cochlear implant, peripheral advantage, central deficit

## Abstract

Individuals deaf from early age often outperform hearing individuals in the visual periphery on attention-dependent dorsal stream tasks (e.g., spatial localization or movement detection), but sometimes show central visual attention deficits, usually on ventral stream object identification tasks. It has been proposed that early deafness adaptively redirects attentional resources from central to peripheral vision to monitor extrapersonal space in the absence of auditory cues, producing a more evenly distributed attention gradient across visual space. However, little direct evidence exists that peripheral advantages are functionally tied to central deficits, rather than determined by independent mechanisms, and previous studies using several attention tasks typically report peripheral advantages or central deficits, not both. To test the general altered attentional gradient proposal, we employed a novel divided attention paradigm that measured target localization performance along a gradient from parafoveal to peripheral locations, independent of concurrent central object identification performance in prelingually deaf and hearing groups who differed in access to auditory input. Deaf participants without cochlear implants (No-CI), with cochlear implants (CI), and hearing participants identified vehicles presented centrally, and concurrently reported the location of parafoveal (1.4°) and peripheral (13.3°) targets among distractors. No-CI participants but not CI participants showed a central identification accuracy deficit. However, all groups displayed equivalent target localization accuracy at peripheral and parafoveal locations and nearly parallel parafoveal-peripheral gradients. Furthermore, the No-CI group’s central identification deficit remained after statistically controlling peripheral performance; conversely, the parafoveal and peripheral group performance equivalencies remained after controlling central identification accuracy. These results suggest that, in the absence of auditory input, reduced central attentional capacity is not necessarily associated with enhanced peripheral attentional capacity or with flattening of a general attention gradient. Our findings converge with earlier studies suggesting that a general graded trade-off of attentional resources across the visual field does not adequately explain the complex task-dependent spatial distribution of deaf-hearing performance differences reported in the literature. Rather, growing evidence suggests that the spatial distribution of attention-mediated performance in deaf people is determined by sophisticated cross-modal plasticity mechanisms that recruit specific sensory and polymodal cortex to achieve specific compensatory processing goals.

## Introduction

Deaf people often show enhanced performance in the visual periphery for certain tasks, like motion detection and spatial localization (Parasnis and Samar, 1985; Bavelier et al., 2006; Pavani and Bottari, 2012), which typically involve auditory–visual integration in hearing people (Dye et al., 2009). This enhancement presumably compensates for the loss of cross-modal auditory information that normally helps individuals to visually orient to unexpected events in their complex changing environment (Parasnis et al., 2003; Bavelier et al., 2006). Neuroimaging and behavioral studies converge to support the hypothesis that peripheral performance enhancements in deaf people are specifically related to population differences in peripheral attentional control (Neville and Lawson, 1987; Bavelier et al., 2000; Bavelier and Neville, 2002). Several studies suggest that in the absence of attentional demands, deaf and hearing people do not differ in performance on peripheral psychophysical tasks like motion processing (Brozinsky and Bavelier, 2004), brightness discrimination (Bosworth et al., 2013), or visual contrast sensitivity (Finney and Dobkins, 2001).

Given prior evidence that peripheral enhancement in deaf people is attention dependent, Proksch and Bavelier (2002) proposed the influential hypothesis that early auditory deprivation alters the gradient of visual attention from the central to peripheral field. Using an interference-from-distraction search task to measure attentional resources, they reported that peripheral distractors interfered with visual search performance more for deaf than hearing participants, whereas central distractors interfered more for hearing participants. Based on these results, Proksch and Bavelier (2002) suggested that early auditory deprivation may expand peripheral attentional resources by drawing resources away from central vision to more equally distribute limited resources across visual space, essentially flattening the gradient of attention in deaf people relative to hearing people. However, they also acknowledged that it remains unclear whether peripheral enhancements and central deficits are linked or are determined by different mechanisms.

The altered gradient of attention proposal is a general hypothesis that offers a neurally plausible model (Pavani and Bottari, 2012) to explain both the peripheral advantages and central deficits reported in the literature across a variety of attention-demanding tasks, irrespective of specific task demands. However, there is currently no definitive evidence to support this proposal as a general mechanism. Prior to and since Proksch and Bavelier’s (2002) study, peripheral enhancements and central deficits in deaf children and adults have been reported by several researchers in a variety of attention-demanding experimental tasks. Typically, peripheral enhancements are found in studies that use dorsal stream tasks (e.g., spatial localization or motion detection) and central deficits are found in studies that use ventral stream tasks (e.g., object identification). However, other than possibly Proksch and Bavelier (2002), we are unaware of any studies that have reported peripheral enhancements concurrently coupled to central deficits, either on the same or different tasks, within the same participants. Typically, studies report peripheral enhancements or central deficits, but not both. Furthermore, one recent divided attention study explicitly designed to test Proksch and Bavelier’s (2002) altered gradient of attention proposal reported a central deficit but no peripheral enhancement (Dye, 2016). Most previous studies were not designed with methodological controls to rigorously test the proposal (e.g., controls for task demands at different eccentricities) and therefore their results have limited evidentiary value.

In the present paper we briefly review the gradient of attention construct and examine the limitations of the existing related literature on early deafness. We then describe the results of an experiment to test the altered gradient of attention proposal using a novel divided visual attention paradigm that overcomes some of the methodological limitations of previous studies. This paradigm involved an object identification task presented in central vision and a concurrent target localization task designed to measure an attentional localization performance gradient from near-central parafoveal locations to peripheral locations. This design allowed us to retain the attention-demanding advantages of a conventional dorsal/ventral stream divided attention task while simultaneously examining the gradient of attention unconfounded by task-specific processing differences. We compared the performance of hearing participants with the performance of prelingually deaf participants with and without cochlear implants (CI) to test specific literature-based predictions of the effect of reduced auditory input on the spatial distribution of attention.

### Gradient of Attention Construct

The gradient of attention construct refers to a continuous decrease in allocation of processing capacity as a function of increasing stimulus eccentricity away from the attended location (LaBerge and Brown, 1989). LaBerge and Brown (1989) defined a formal model that incorporates both a fixed structural acuity gradient and an independent attentional gradient that can be dynamically reshaped under different task demands to alter perceptual performance across the visual field. For example, their model allows for the center of attention to move away from fixation depending on task demands. However, in tasks involving unguided attention to locations symmetric about fixation and central processing demands or foveal loads, as is typical of many studies of enhanced peripheral attention in deaf people, the center of vision is generally the focus of attention (LaBerge and Brown, 1989; Staugaard et al., 2016).

Previous research on hearing people supports this model. Spatial attention is generally resource limited, and attentional gradients diminish continuously from the focus of attention, with the spread of the gradient adjusting to match the range of possible target locations in distributed attention tasks (see Bush, 2012, for a review). Consistent with the proposal that early auditory deprivation drives a more equal distribution of attentional resources toward the periphery, experimental conditions that require people to spread their attention over a wider spatial range under exactly the same task requirements cause them to move some attentional resources away from the center of attention, altering the availability of attentional resources everywhere within the range, including at and near the center of attention (Greenwood and Parasuraman, 1999; Bush, 2012), thereby flattening the slope of the gradient.

Most recently, Staugaard et al. (2016) used Bundesen’s (1990) Theory of Visual Attention to confirm that several independently estimable components of attention (visual short term memory capacity, visual perceptual threshold, visual processing speed) diminish monotonically with increasing target eccentricity away from central vision, independent of visual system structural gradients like the cortical magnification factor and of eccentricity dependent motor reactions. Staugaard et al. (2016) reported further that manipulating endogenous attention did not alter these attentional gradients, but cited Proksch and Bavelier’s (2002) study to support the claim that long-term environmental factors may lead to a compensatory trade-off between attentional resources in peripheral vs. central vision.

### Early Deafness and the Gradient of Attention

Although some behavioral and neurophysiological studies of visual performance in deaf adults and children are consistent with an experience-driven altered general gradient of attention proposal, other than possibly Proksch and Bavelier (2002), studies have not demonstrated that peripheral enhancements are concurrently linked to central deficits as a limited attentional resource model would predict. Peripheral enhancements without concurrent central deficits have been shown in several behavioral and neuroimaging studies (e.g., Neville and Lawson, 1987; Loke and Song, 1991; Bavelier et al., 2000). Conversely, Dye (2016) showed central deficits on a divided visual attention task but equivalent concurrent peripheral performance. Similarly, Sladen et al. (2005) showed that deaf adults display greater interference than hearing adults from incompatible flankers at a parafoveal location, but equal interference at a central location, suggesting that a broader spread of visual resources in the deaf group was not accompanied by a central deficit in visual resources. Parasnis and Samar (1985) showed enhancement for reorienting to a peripheral stimulus when central stimuli compete for attention, and Shiell et al. (2014) showed peripheral enhancement for visual motion detection thresholds, but neither study tested performance centrally. Other attention studies have shown central deficits on continuous performance tests (CPT), but they did not test at peripheral locations to confirm an attentional tradeoff (Quittner et al., 1994; Mitchell and Quittner, 1996; Smith et al., 1998; Parasnis et al., 2003; Horn et al., 2005). Still other CPT studies failed to show central deficits for deaf children (Tharpe et al., 2002; Dye and Hauser, 2014). Bosworth and Dobkins (2002) showed no reliable peripheral advantages or central deficits for coherent motion detection thresholds for deaf participants, even when attention was cued to the target stimulus location. Thus, no consistent picture of concurrently coupled peripheral advantages and central deficits associated with auditory deprivation has emerged from the literature. Generally, attention studies comparing deaf and hearing participants have varied widely in experimental design, task demands, eccentricity of stimuli, and participant deaf group characteristics (e.g., chronological age, age of onset and etiology of deafness, CI use, controls for medical or developmental conditions). Hence, methodological limitations and participant group differences could potentially account for the lack of consistent results across studies (Bosworth and Dobkins, 2002; Dye et al., 2009).

### Divided Attention Studies and the Gradient of Attention

Previous research collectively indicates that compensatory changes in the distribution of attention across the visual field associated with auditory deprivation are best revealed by attention-dependent paradigms involving competing central and peripheral tasks, uncertainty about target location, and the presence of distractor stimuli (Bavelier et al., 2006; Dye and Bavelier, 2013), all conditions typical of real world environments. Experimentally, these conditions are most closely approximated in divided selective attention paradigms that require participants to perform a central ventral stream task (e.g., an object identification task) and a concurrent peripheral dorsal stream task (e.g., a localization or movement detection task) in the presence of spatially distributed distractors. Furthermore, studies have shown that performance on divided attention tasks, especially those that engage working memory and involve central identification tasks and peripheral localization or motion detection tasks, predict real-world daily life performance in a number of normal and clinical populations (Clay et al., 2005; Miloyan et al., 2013). Since the peripheral advantage in deaf people has been generally regarded as an adaptation to compensate for the loss of auditory information in real world settings (Parasnis, 1983; Parasnis et al., 2003; Bavelier et al., 2006; Pavani and Bottari, 2012), we would expect divided attention paradigms involving central ventral stream tasks and peripheral dorsal stream tasks to provide an ideal laboratory protocol for testing the altered gradient of attention proposal.

Few previous studies have searched for peripheral advantages and central deficits using divided attention paradigms. Bosworth et al. (2013) used a divided attention paradigm to compare deaf and hearing adults on static stimulus orientation discrimination and motion perception performance tasks within central and peripheral regions while participants concurrently counted target shapes in a central RSVP task. They reported no peripheral advantages or central deficits for either orientation or motion tasks. Importantly, the performance of deaf subjects on the central RSVP task was significantly worse than the performance of hearing subjects regardless of whether the RSVP stimuli occurred during the motion or orientation tasks at peripheral or central locations. Thus, these results reveal a selective central deficit for object detection (a ventral stream task) in deaf adults compared with hearing adults in a divided attention paradigm, but equivalent group performance for detecting motion (a dorsal stream task) as well as for discriminating static orientation (a competing ventral stream task) at both central and peripheral locations. These results suggest that attentional control of central task performance may be independently determined by specific task demands rather than governed by a redistribution of general attentional resources across space. Therefore, this result does not support the altered gradient of attention proposal.

Dye et al. (2009) tested deaf and hearing adults and children on the Useful Field of View (UFOV) test, a divided selective attention paradigm that requires participants to localize a peripheral target among distractors and concurrently discriminate the identity of a central target. Deaf adults had shorter peripheral stimulus duration thresholds than hearing adults on the UFOV, but not on a simpler divided attention task not involving distractors. Dye et al. (2009) attributed these UFOV results to enhanced attention to peripheral stimuli due to auditory deprivation. However, they only measured peripheral thresholds on trials where both central identification and peripheral localization were correct, and did not independently identify thresholds at the central site. Consequently, relative group performance on the central identification task was unknown. Therefore, as Dye (2016) acknowledged, the performance enhancement observed in Dye et al. (2009) cannot be linked to a shift in the gradient of attention for deaf participants involving selective peripheral enhancement and concurrently deficient central performance.

Dye (2016) disentangled participants UFOV performance on the central identification and peripheral localization tasks by measuring separate peripheral and central thresholds concurrently to specifically test Proksch and Bavelier’s (2002) altered gradient of attention proposal. Dye reported a deficit on the central identification task for deaf adults that only appeared under attentionally demanding competition from peripheral targets and distractors. However, contrary to expectation and to the previous results of Dye et al. (2009), deaf and hearing adults did not differ on peripheral performance. Thus, under demanding conditions of divided attention between central identification and peripheral localization tasks, deaf adults did not display superior peripheral performance despite an apparent reduction of attentional resources in central vision, suggesting, like Bosworth et al.’s (2013) results, that the central deficit might have been specific to Dye’s (2016) foveated ventral stream object identification task.

### The Divided Gradient of Attention Paradigm (DGAP)

Dye’s (2016) results converge with those of Bosworth et al. (2013) to suggest that peripheral advantages and central deficits in previous studies may have been caused by independent mechanisms rather than by an altered general gradient of attention. However, both of their paradigms have methodological limitations for testing the altered gradient of attention proposal. In general, the UFOV paradigm that Dye (2016) used completely confounds ventral and dorsal stream task demands with central vs. peripheral stimulus location, respectively. Because object identification and target localization are mediated by distinct ventral and dorsal stream mechanisms (Ungerleider and Haxby, 1994; Weisberg et al., 2012), a flattening of the performance gradient due either to a peripheral advantage alone, a central deficit alone (as in Dye), or even both concurrently, cannot distinguish between a redistribution of general attentional resources from central to peripheral locations on the one hand, and population differences in underlying neural control of specific task performance. Bosworth et al.’s (2013) paradigm does nominally overcome this confound in that their orientation and motion stimuli were presented at both central and peripheral locations while participants concurrently performed a foveated identification task. However, their central and peripheral motion and orientation stimuli subtended a full 5° of visual angle within three immediately adjacent regions that spanned a total of only 7.5° on either side of fixation. Therefore, participants’ performance at the study’s nominal peripheral locations actually integrated across broad regions of parafoveal space that were closely situated to the nominal central location which, itself, integrated across foveal and parafoveal regions. This design cannot provide sufficiently high resolution to measure performance at discrete, well separated locations from central to peripheral regions across the attentional gradient.

**FIGURE 1:**
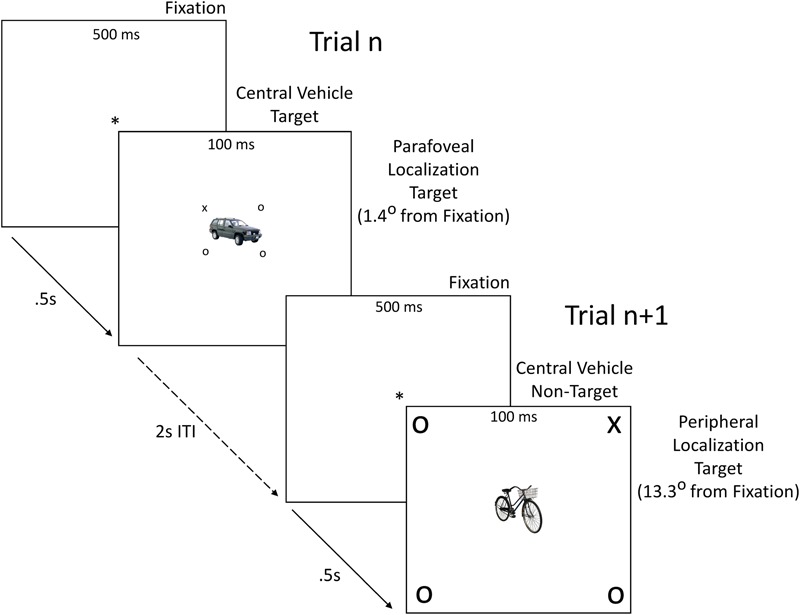
**Sample stimuli and trial structure.** On each trial, a fixation point (^∗^) lasting 500 ms was replaced by a central vehicle target or non-target flanked by a localization target (X) and three distractors (O) for 100 ms, followed by a blank screen for an inter-trial interval (ITI) of 2 s. Central vehicle targets were either cars or non-cars. Probability of a central vehicle target or non-target appearing on a given trial was 0.5. Probability of a localization target X appearing at any one of the eight parafoveal or peripheral locations on a given trial was 0.125. Vehicle category (car vs. non-car) was perfectly balanced across localization target location. Sample vehicle images are taken from the POPORO set (Kovalenko et al., 2012). Stimuli in figure are not drawn to scale.

In the present study, we employed a new divided attention paradigm to test the altered gradient of attention proposal that preserves the ecologically sensible combination of a central identification task and an eccentric localization task while simultaneously dissociating central task performance from performance along the gradient of attention for the localization task. Participants performed a vehicle identification task that required them to distinguish cars from other vehicles on each trial and keep track of how many target vehicles they saw across a block of trials (**Figure 1**). The central identification task included many distinct vehicle exemplars and a sustained working memory component, establishing a demanding level of competition with the localization task. Participants simultaneously identified the location of a target stimulus (X) among symmetrically placed distractors (circles) presented unpredictably at either near-central parafoveal (1.4° from fixation) or peripheral (13.3° from fixation) locations (**Figure 1**). Previous studies have reported attentional advantages for deaf participants over a broad range of eccentricities from 2° to more than 20° (Parasnis and Samar, 1985; Pavani and Bottari, 2012). Our use of a near-central parafoveal location less than 1.5° from fixation, and a peripheral location well into the range of previously reported peripheral advantage effects, allowed us to examine the linear slope of the localization gradient between these two discrete points for deaf and hearing groups when attention was spread broadly over a considerable angular distance, independent of their central identification task performance.

### Hypotheses

We developed two related hypotheses for performance on the DGAP to test the altered gradient of attention proposal. Hypothesis 1 is that deaf participants without CIs will show a set of related effects, namely worse central identification performance, better peripheral localization performance, and a flatter parafoveal-peripheral response gradient in the localization task, relative to hearing participants. This hypothesis is directly implied by the previous literature on attentional gradients and the altered gradient of attention proposal. Confirmation of only the central identification deficit would not support the altered gradient of attention hypothesis, but would be consistent with the alternative hypothesis that the central identification deficit is independent of attentional effects on competing tasks at other eccentricities.

Hypothesis 2 is contingent on confirmation of Hypothesis 1. Assuming Hypothesis 1 is supported for deaf participants without CIs, we predict a selective pattern of performance for deaf participants with CIs. Specifically, we hypothesize that deaf participants with CIs will not show inferior central identification task performance, superior peripheral localization performance, or a flatter localization gradient than the hearing group. This hypothesis is motivated by previous CPT studies that report that deaf children without CIs show central attentional deficits, but deaf children with CIs show central attentional skills that approach those of hearing children by about 18 months after implantation (Quittner et al., 1994; Smith et al., 1998). Accordingly, consistent with the altered gradient of attention proposal, previous authors have speculated that CI users, unlike CI non-users, should not display enhanced target or motion detection in the periphery since they can use auditory cues to support cross-modal integration (Kim et al., 2016). Note that if Hypothesis 1 is not supported, confirmation of Hypothesis 2 offers no support for the altered gradient of attention hypothesis since non-inferiority of performance would not then be selectively associated with restored auditory input.

The altered gradient of attention hypothesis also predicts that the hearing-typical attention gradient should be restored in CI users as a function of time-with-implant. Specifically, across CI-implanted individuals, we would expect increased time-with-implant to be associated with reduced central attentional deficits and with contemporaneously reduced peripheral advantages due to newly restored auditory experience. However, limited research on deaf children has failed to find a correlation between time-with-implant and central attentional performance (Smith et al., 1998). No one has tested the relationship in any attention-dependent tasks in deaf adult CI users. Therefore, we explored this relationship for our two tasks to determine whether it conformed to the predictions of the altered gradient of attention hypothesis.

Most previous studies have not screened their deaf or hearing participants for known or hidden attention and executive function deficits that can influence group performance differences in attention studies. Considering that ADHD is a high prevalence, often hidden disorder in all groups and especially in the deaf population (Samar et al., 1998), this is a significant methodological shortcoming that we address in two ways. First, we tested only participants who reported no history of ADHD diagnosis. Second, we administered the Behavior Rating Inventory of Executive Functions–Adult Form (BRIEF-A) to all participants to control for variation in executive function and its signature disorder, ADHD. We have previously validated the BRIEF-A for use with deaf adult college students, and have shown that it is sensitive to the presence of ADHD in this population (Hauser et al., 2013).

## Materials and Methods

### Participants

Twenty-five deaf and 25 hearing students at the Rochester Institute of Technology were recruited through flyers and RIT’s on-line experiment participation system and were paid $10 and/or given psychology course participation credit. They were screened for self-reported history of ADHD diagnosis, neurological disorders, severity and age of onset of hearing loss, and vision problems. Seven deaf and three hearing participants were eliminated because of a history of Usher Syndrome, neurological disorders or illness, ADHD diagnosis, or becoming deaf after 3 years old. The remaining 22 prelingually deaf young adults, 11 with cochlear implants (CI group) and 11 without cochlear implants (No-CI group), and 23 hearing young adults were included in the study.

**Table 1** presents psychometric and demographic characteristics for these groups. The groups had comparable overall scores on the BRIEF-A near the normative population mean (*T*-score = 50) and all individual subjects scored within approximately 1.6 SD of the mean, indicating no evidence of attentional or other executive function disorder in any group. Gender composition across groups was significantly different. The two deaf groups had somewhat different distributions of previous school types but were otherwise comparable. All deaf participants reported early severe to profound hearing loss, however, audiometric hearing loss data were not available.

**Table 1:** Participant demographics by group.

General demographics				
**Measure**	**Level**	**Group**					
		**CI**		**No-CI**		**Hearing**	
		***M***	***SD***	***M***	***SD***	***M***	***SD***

BRIEF-A (*T*-scores)	BRI	52.4	5.1	51.5	6.3	53.9	6.4
	MI	55.1	6.0	52.5	7.9	52.6	6.2
	GEC	53.9	4.8	52.1	7.0	53.2	5.7
Age (years)		21.1	1.6	20.3	1.7	19.5	3.3
		
		**%**	***N***	**%**	***N***	**%**	***N***

Gender^∗^	Female	36.4	4	72.7	8	21.7	5
	Male	63.6	7	27.3	3	78.3	18
Hispanic ethnicity	Hispanic	18.2	2	0	0	8.7	2
	Non-hispanic	81.8	9	100	11	21	2
Race	White	81.8	9	90.9	10	69.6	16
	Black/African American			9.1	1	8.7	2
	Asian	9.1	1			13.0	4
	Other	9.1	1			8.7	2
Childhood SES	Low	9.1	1	9.1	1	17.4	4
	High	90.9	10	90.9	10	82.6	19

**Deaf demographics**				

**Measure**	**Level**	**Group**						
		**CI**		**No-CI**				
		***M***	***SD (Range)***	
		
Age of cochlear implant surgery (months)		78.5	52.2 (18–204)
Time-with-implant (months)		174.5	51.5 (48–252)
		
		***%***	***N***	***%***	***N***		

Cultural identity	Culturally deaf	36.4	4	54.6	6		
	Deaf	27.3	3	27.3	3		
	Hard of hearing	18.2	2	18.2	2		
	Oral deaf	18.2	2				
Age of hearing loss	Birth	81.8	9	81.8	9		
	Before 3 years	18.2	2	18.2	2		
Parents hearing status	One or both deaf	9.1	1	36.4	4		
	Hearing	90.9	10	63.6	7		
Best language	ASL/Sign language	18.2	2	45.5	5		
	English	27.3	3	9.1	1		
	Both	54.5	6	45.5	5		
School types^∗^	Schools for the deaf			36.4	4		
	Mainstreamed	72.7	8	27.2	3		
	Both	27.3	3	36.4	4		

*^∗^Group comparison χ^2^*p* < 0.025.*

### Measures

#### Behavior Rating Inventory of Executive Functions–Adult Form (BRIEF-A, Roth et al., 2005)

The BRIEF-A is a 75-item self-report instrument with nine clinical scales: Inhibit, Shift, Emotional Control, Self-Monitor, Initiate, Working Memory, Plan/Organize, Task Monitor, and Organization of Materials- The instrument yields a Behavioral Regulation Index (BRI), a Metacognitive Index (MI), and a Global Executive Composite (GEC) that provides an overall measure of executive function. Hauser et al. (2013) have demonstrated psychometrically that the BRIEF-A is a reliable, unbiased diagnostic tool for use with deaf college students, with discriminant and predictive validity for ADHD diagnosis comparable to that for the hearing college population.

#### Divided Gradient of Attention Paradigm (DGAP)

The DGAP consists of a central object identification task and a concurrent spatial localization task. The object identification task required participants to identify centrally presented vehicles as belonging to the class of cars or non-cars (other vehicles). Either cars or non-cars were designated as target vehicles and the other vehicle set as non-targets, counterbalanced across participants (**Figure 1**). At the end of each of four 80-trial blocks, participants reported how many target vehicles appeared during that block. The concurrent spatial localization task required participants to press a button to indicate whether an X target appeared on the left or right of fixation. On each trial, along with the central vehicle, the X appeared at one of eight locations, either parafoveally near the center of the visual field or peripherally, simultaneous with three symmetrically placed circle distractors (**Figure 1**). Reporting which side of fixation the target occurred instead of its specific location ensured identical motor responses for parafoveal and peripheral stimuli.

The central vehicle identification task was attentionally challenging, requiring participants to selectively attend to local stimulus features that define vehicle category membership over a large range of vehicle exemplars and to hold a running sum in working memory. Additionally, the large number of vehicles helped prevent participants from overlearning the vehicle stimulus set and therefore helped maintain sustained attention for local defining features near the center of vision.

The vehicles were selected from standard stimulus sets (Kovalenko et al., 2012; Moreno-Martínez and Montoro, 2012), and a few needed additional car stimuli were randomly acquired from arbitrary internet sites. A variety of car models, colors, and styles (e.g., sports car, SUV, sedan, convertible) and non-car vehicles (e.g., train, bicycle, skateboard, hot air balloon, rocket, plane, wagon, truck, baby carriage) were included. Twenty-four cars and 24 non-car vehicles were selected for a total of 48 distinct vehicles. The vehicles spanned a region of 2° centered at fixation on the monitor display.

The X target stimulus and the circles were symmetrically placed along the inter-cardinal directions at 45, 135, 225, and 315°. Parafoveal targets and distractors spanned 0.23°, centered 1.4° from fixation immediately adjacent to the vehicle. Peripheral targets and distractors spanned 0.77°, centered 13.3° from fixation. The size of the localization stimuli was adjusted to compensate for the cortical magnification factor (Virsu and Rovamo, 1979).

To construct the final stimuli, the orders of the 24 cars and the 24 non-cars were separately randomized. The first five cars and the first five non-cars were paired with an X appearing on the upper left inter-cardinal line at 1.4° from center (e.g., **Figure 1**, Trial *n*). Similarly, the next three sets of five cars and five non-cars were paired with X’s appearing on the upper right, lower right, and lower left inter-cardinal lines, respectively. Circle stimuli were placed symmetrically on the remaining inter-cardinal lines. This procedure resulted in 20 unique cars and 20 unique non-cars paired with parafoveal X (target) stimuli equally distribute over the four inter-cardinal positions. The same vehicle and target-position stimulus pairings were then reproduced with the peripheral X’s to create a parallel set of 40 target stimuli equally distribute over the four peripheral inter-cardinal positions (e.g., **Figure 1**, Trial *n*+1). Thus, across these 80 stimuli, the attentional and processing demands associated with specific central vehicle images were perfectly matched between parafoveal and peripheral target sets.

These 80 stimuli were presented as the first and forth block of the four-block experimental session, in a different trial random order, with a total vehicle-class target count of 40 and non-target count of 40 for each of those two blocks. To avoid participants learning the expected vehicle target count per block after the first block, the number of target vehicles was reduced to 32 and the number of non-target vehicles was increased to 48 for block 2. Conversely, for block 3 the number of target and non-target vehicles was increased to 48 and reduced to 32, respectively. To reduce the number of target vehicles in block 2, four randomly chosen stimuli containing a target vehicle and an X at each of the four parafoveal positions and the corresponding four stimuli containing the same four target vehicles and X at each of the four peripheral positions were removed. To increase the number of non-target vehicles in block 2, the four unused non-target vehicles from the appropriate original 24 vehicle set were each paired with one parafoveal X and one peripheral X at one of the four inter-cardinal directions. This procedure maintained the equal distribution of parafoveal and peripheral locations and vehicle pairings within each vehicle-class stimulus set. For block 3, the corresponding procedure created the 48 target vehicle stimuli using the four unused target vehicles and reduced the number of non-target vehicle stimuli to 32. This procedure resulted in a completely balanced set of 320 stimuli across four blocks of 80 stimuli each. To counterbalance cars and non-cars as target vehicles, two sets of 320 stimuli were constructed using the appropriate target and non-target vehicles across all four blocks. Thus, half the participants counted cars as target vehicles with a distribution of 40, 32, 48, and 40 car counts across the four blocks and half counted non-cars as target vehicles with a distribution of 40, 32, 48, and 40 non-car counts across the four blocks.

### Procedure

This study was carried out in accordance with the recommendations of the RIT Human Subjects Research Office with written informed consent from all subjects. All subjects gave written informed consent in accordance with the Declaration of Helsinki. The protocol was approved by the RIT Human Subjects Research Office.

#### Demographic and Psychometric Testing

All participants were screened for a history of ADHD, neurological disorders, and vision problems, and then responded to a computerized survey to report the demographic and deaf demographic information in **Table 1**. Participants whose both parents had high school or vocational degrees or lower were classified as low childhood socioeconomic status. Participants who had one or more parents with some college attendance or higher degrees were classified as high childhood socioeconomic status. Additionally, deaf participants reported their deaf cultural identity, age of onset of hearing loss, best language, types of schools attended, and parents’ hearing status. Participants took the BRIEF-A immediately after the demographic survey.

#### Experimental Protocol

Participants sat 58″ from a SONY GDM-F500 21in monitor. The vehicle identification and spatial localization tasks were introduced separately in a practice session. Participants first saw a practice sequence of 12 cars and non-cars, flashed in the center of the screen. A fixation asterisk appeared for 500 ms, followed by the vehicle for 100 ms, followed by a 2 s inter-trial interval (ITI). Each participant was assigned either cars or non-cars as target vehicle, and reported their target count at the end of this practice block. After a minute rest, participants saw a practice sequence of 12 spatial target trials, with an inter-trial interval of 2 s, containing a fixation asterisk for 500 ms followed by an X and three symmetrically placed circles presented for 100 ms at either the parafoveal or peripheral location, but without a central vehicle. Participants practiced pressing a button with their right or left index finger to indicate the appearance of the X on the right or left of fixation, respectively. After another minute rest, the two tasks were combined for a third practice run of 12 trials, and participants responded to the X’s with a button press on each trial and simultaneously kept track of the count of their target vehicles to report after the practice block.

Participants then completed the four blocks of 80 trials with a minute rest between blocks. Response times (RT) and accuracy on each trial were recorded for the localization task. Participants used a keyboard to enter their total target vehicle count at the end of each 80-trial block. They were told to try to be accurate in their vehicle count and to be both fast and accurate in localizing the X’s.

#### Analysis

For the central vehicle identification task a total percent correct accuracy score was computed for each participant as 100 minus the absolute value of the percent difference score, where percent difference score = 100^∗^((vehicle count reported)–(vehicle count presented))/(vehicle count presented). This measure takes account of the fact that some participants reported fewer and some reported more than the total number of target vehicles that were presented. Like ordinary percentages, a 100% score means perfect performance. An 85% score means that the participant either incorrectly identified 15% of the targets as non-targets or 15% of the non-targets as targets, and so on. For the target localization task, the percent correct localization accuracy for target stimuli presented at each of the eight parafoveal and peripheral locations was computed as the number correct out of the total number of targets presented at that location across all four blocks. A total parafoveal localization accuracy score was computed as the average of the percent accuracy across the four parafoveal locations. Similarly, a total peripheral localization accuracy score was computed as the average of the percent accuracy across the four peripheral locations. The percentage scores were arcsine transformed to correct for the inherent deviation from normality of percentage scores.

Trimmed mean correct RTs were computed for parafoveal and peripheral locations. For each participant, any trial RT exceeding 2 standard deviations around their mean of all correct trials within a given block was scored as an error and dropped from the analysis. Trimmed mean correct RTs were then computed for each of the eight locations across all four blocks. Parafoveal and peripheral localization mean RT’s were then computed as the average of the four mean RTs at their respective eccentricities.

Preliminary correlational analyses were conducted to rule out the presence of statistically reliable speed-accuracy tradeoffs. Speed-accuracy correlations were computed both within and across tasks for each group separately.

We used planned comparisons to test the predictions of our hypotheses. Planned comparisons offer greater statistical power than unplanned omnibus tests, such as ANOVA, to test group differences when specific hypotheses based on predictions from the literature are planned in advance, and have been used in previous divided attention work to test the altered gradient of attention hypothesis (Dye, 2016). Accordingly, based on clear predictions from previous studies that report central deficits in stimulus identification for non-CI users (Quittner et al., 1994; Mitchell and Quittner, 1996; Smith et al., 1998; Parasnis et al., 2003; Horn et al., 2005; Dye, 2016), but not CI users (Quittner et al., 1994; Smith et al., 1998; Horn et al., 2005) we used a directional planned pairwise comparison to test the prediction from Hypothesis 1 that the No-CI group would perform worse than the hearing group on the central identification task. We also compared the central identification task performance of the CI group against that of the hearing group to test the prediction from Hypothesis 2 of non-inferiority for that group.

Since a peripheral advantage for the No-CI group is expected based on much previous literature (Bavelier et al., 2006; Pavani and Bottari, 2012), we conducted directional planned pairwise comparisons to test the prediction from Hypothesis 1 that the No-CI group would perform better than the hearing group on localization accuracy and localization RT at the peripheral location. We also compared the peripheral performance of the CI group against that of the hearing group as well to test the prediction from Hypothesis 2 of non-superiority for that group.

Hypothesis 1 also predicts that the parafoveal-peripheral localization gradient would be flatter for the No-CI group than for the hearing group. This is a specific form of interaction between these two groups and the two localization eccentricities (parafoveal, peripheral). We were not interested in testing all possible group by eccentricity interactions, only the predicted one. Therefore, we computed parafoveal-peripheral difference scores within participants to represent the gradient and then compared the No-CI group with the hearing group on these difference scores using a directional planned comparison following our prediction. A significant group difference in the direction of smaller parafoveal-peripheral difference scores for the No-CI group would support the specific predicted interaction in Hypothesis 1 of a flatter localization gradient. We examined the parallel interaction for the CI and hearing groups to test the prediction from Hypothesis 2 of no flatter localization gradient for the CI group.

For group and correlational analyses, we conducted both unadjusted comparisons and follow-up adjusted comparisons including age, gender, race/ethnicity (coded as white non-Hispanic vs. other race/ethnicity), and childhood SES as covariates. These common demographic variables are known to affect performance in object identification and localization tasks in particular and can be a significant source of artefactual findings in attentional studies using convenience samples (Scialfa et al., 1994; Hackman and Farah, 2009; McGugin et al., 2012; Clearfield and Jedd, 2013; McKone et al., 2013; Gruber et al., 2014). They have generally not been controlled in previous studies. Including both unadjusted and adjusted analyses allowed us to confirm that group differences in performance on the identification and localization tasks were not confounded by group sampling differences on these known demographic variables. In addition, to rule out possible general perceptual-motor processing differences among groups as an explanation for group differences within specific tasks, we included analyses that adjusted group performance on the central identification task for overall group performance differences on the localization task, and vice-versa. We also included analyses adjusted for BRIEF-A GEC scores to further control the potential influence of group or individual variation in overall executive function related to deafness (Hintermair, 2013). Finally, much evidence suggests that deaf and hearing groups may differ in their working memory skills (Hall and Bavelier, 2010). Because our central identification task relied heavily on working memory, we also conducted analyses that specifically controlled for group and individual differences in working memory using the BRIEF-A Working Memory subscale.

## Results

Accuracy for the two tasks was generally high and well distributed for all groups, indicating that the groups successfully performed both tasks: central identification task, *M*(*SD*), No-CI: 84.7%(16.9%), CI: 88.3%(15.9%), Hearing: 94.1%(5.1%); localization task *M*(*SD*), No-CI: 95.4%(3.1%), CI: 93.0%(9.0%), Hearing: 94.9%(3.7%). There were no significant or marginal correlations between RT and accuracy within the localization task at parafoveal or peripheral sites, overall or for any group individually. Similarly, accuracy in the central identification task was not related to RT at parafoveal or peripheral sites overall or for any group. Thus, there was no evidence that any group differences in accuracy or RT could be attributed to a speed-accuracy tradeoff.

### Accuracy Group Comparisons

#### Central Identification Accuracy Group Comparisons

**FIGURE 2:**
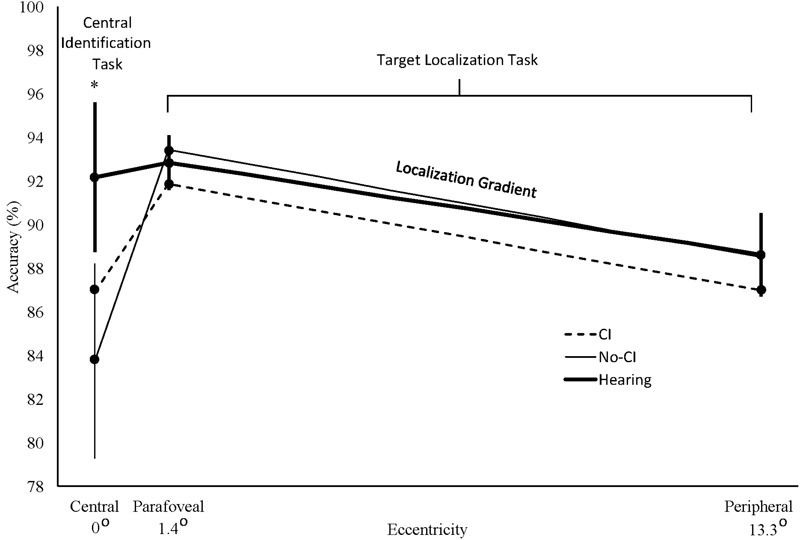
**Percent accuracy for central vehicle identification task targets (centered at 0° eccentricity) and for parafoveal (1.4°) and peripheral (13.3°) localization task targets.** Localization gradients for each group are represented by the straight lines connecting the data points between parafoveal and peripheral locations. These lines represent the piecewise slope determined by sparse sampling at two discrete eccentricities along the performance gradients for each group. They are not intended to imply accurately interpolated values at intermediate eccentricities that were not sampled in our study or to reflect any assumption that the attentional gradient between those sampled eccentricities is strictly linear for any group. CI: deaf cochlear implant group; No-CI: deaf group without cochlear implants; Hearing: hearing group. Error bars are standard errors for the hearing group (thick bars) and for the No-CI group (thin bars). ^∗^No-CI vs. Hearing Group planned comparison, *p* < 0.035.

As predicted by previous studies and Hypothesis 1, the unadjusted planned comparisons showed that the No-CI group performed significantly worse than the hearing group on the central identification task, No-CI: *M* = 84.7%, *SE* = 3.6; Hearing: *M* = 94.1%, *SE* = 2.5; *t*(42) = -2.1, *p* = 0.0195. By contrast, the CI group mean was numerically intermediate between the No-CI and hearing group means and was not significantly inferior to the hearing group mean, CI: *M* = 88.3%, *SE* = 3.6; Hearing: *M* = 94.1%, *SE* = 2.5; *t*(42) = -1.1, *p* = 0.1412. Adjusting for age, gender, race/ethnicity, and childhood SES produced the same results as the unadjusted analysis, No-CI: least squares *M* = 83.7%, *SE* = 4.5%; Hearing: least squares *M* = 92.2%, *SE* = 3.4%; *t*(38) = -1.9, *p* = 0.0327, and CI: least squares *M* = 86.9%, *SE* = 4.5%; Hearing: least squares *M* = 91.9%, *SE* = 3.4%; *t*(38) = -1.1, *p* = 0.1368. **Figure 2** displays the adjusted least squares group means for the central identification task, plotted at 0° on the eccentricity axis. The same central deficit for the No-CI group was still obtained when the three groups were further equated on parafoveal localization accuracy, *t*(37) = -2.2, *p* = 0.0177, peripheral localization accuracy, *t*(37) = -2.2, *p* = 0.0184, or overall localization accuracy (average of parafoveal and peripheral accuracy), *t*(37) = -2.4, *p* = 0.0110, confirming that significantly worse performance of the No-CI group was not due to overall worse performance on both tasks together. The CI group continued to show no significant inferiority to the hearing group on the central identification task in analyses adjusted for parafoveal localization accuracy: *t*(37) = -0.9, *p* = 0.1761, peripheral localization accuracy, *t*(37) = -0.9, *p* = 0.1891, or overall localization accuracy (average of parafoveal and peripheral localization accuracy), *t*(37) = -0.8, *p* = 0.2094. In addition, the central deficit remained for the No-CI group after controlling further for the BRIEF-A GEC scores, *t*(36) = -2.2, *p* = 0.0189, as well as for BRIEF-A working memory scale scores, *t*(36) = -2.4, *p* = 0.0103, indicating that this deficit was not accounted for by group differences in overall executive functions or working memory in particular. The CI group continued to show no significant inferiority to the hearing group on the central identification task after controlling for BRIEF-A scores, *t*(36) = -1.1, *p* = 0.1360, as well as for BRIEF-A working memory scale scores, *t*(36) = -1.0, *p* = 0.1547.

To confirm that the central deficit on the object identification task in the planned comparisons for the No-CI group was robust against possible unknown violations of distributional assumptions due to small sample size, we conducted follow-up distribution free directional Wilcoxon two-sample tests. The results matched those of the planned comparisons, confirming the central identification deficit for the No-CI group and no significant deficit for the CI group: No-CI: *z* = -1.9, *p* = 0.0286, and CI: *z* = -0.3, *p* = 0.3769.

#### Peripheral Localization Accuracy Group Comparisons

Contrary to the prediction of Hypothesis 1, unadjusted planned comparisons of the No-CI group against the hearing group revealed no accuracy advantage at the peripheral location, *t*(42) = -0.3, *p* = 0.6150. Adjusting for age, gender, race/ethnicity, and childhood SES produced the same results as for the unadjusted analysis, *t*(38) = -0.03, *p* = 0.5110. **Figure 2** displays the adjusted least squares group means for analysis of the localization task at the peripheral location, plotted at 13.3° on the eccentricity axis. Further adjusting for central identification accuracy did not cause a No-CI group advantage to emerge *t*(37) = -1.0, *p* = 0.8305. In addition, no peripheral advantage emerged for the No-CI group after controlling further for BRIEF-A GEC scores, *t*(36) = 1.8, *p* = 0.1227, as well as for BRIEF-A working memory scale scores, *t*(36) = 1.3, *p* = 0.1064, indicating that the lack of superior peripheral performance was not accounted for by group differences in overall executive functions or working memory in particular. Planned comparisons of the CI group against the hearing group also revealed no reliable performance superiority at peripheral locations in unadjusted analyses, *t*(42) = -0.6, *p* = 0.7372. Nor were there any reliable group superiorities in analyses adjusted for the demographic measures, *t*(38) = -0.4, *p* = 0.6469, nor when further adjusted for central identification accuracy, *t*(37) = -0.2, *p* = 0.5708, nor after controlling for BRIEF-A GEC scores, *t*(36) = 0.1, *p* = 0.4437, as well as for BRIEF-A working memory scale scores, *t*(36) = -0.02, *p* = 0.5065. **Figure 2** shows that the No-CI and hearing group peripheral localization task means were nearly identical, and all group means were within one standard error of the hearing group mean.

#### Localization Accuracy Gradient Group Comparisons

Contrary to the prediction of Hypothesis 1, unadjusted planned comparisons of the No-CI group against the hearing group for the parafoveal-peripheral localization accuracy difference scores revealed no group differences in their performance gradients, *t*(42) = 1.2, *p* = 0.8861. The same result was obtained after adjusting for demographic measures, *t*(38) = 0.6, *p* = 0.7092. The localization task gradient for each group is defined by the line connecting the means between 1.4 and 13.3° in **Figure 2**. The figure shows that the No-CI group’s localization task gradient was not flatter than the hearing group’s gradient. Further adjusting for central identification accuracy did not cause a flatter gradient to emerge for the No-CI group, *t*(37) = 0.1, *p* = 0.5380. In addition, no reliably flatter gradient emerged for the No-CI group after controlling further for BRIEF-A GEC scores, *t*(36) = -0.1, *p* = 0.4663, as well as for BRIEF-A working memory scale scores, *t*(36) = -0.2, *p* = 0.4033, indicating that the lack of superior peripheral performance was not accounted for by group differences in overall executive functions or working memory in particular. Planned comparisons of the parafoveal-peripheral localization accuracy difference scores for the CI group against the hearing group also revealed no reliably flatter gradient in unadjusted analyses, *t*(42) = 0.4, *p* = 0.6692. Nor was there any reliably flatter gradient in analyses adjusted for the demographic measures, *t*(38) = 0.2, *p* = 0.5736, nor when further adjusted for central vehicle identification accuracy, *t*(37) = -0.1, *p* = 0.4680, nor after controlling for BRIEF-A GEC scores, *t*(36) = -0.04, *p* = 0.4826, as well as for BRIEF-A working memory scale scores, *t*(36) = 0.1, *p* = 0.5570. **Figure 2** shows that, like the group peripheral localization task means, the group parafoveal localization task means were all within one standard error of the hearing group mean. A full set of analyses on the parafoveal accuracy data alone, parallel to those performed for the peripheral accuracy data above, showed no significant group differences in unadjusted or adjusted analyses at the parafoveal locations. Therefore, as these analyses show, contrary to Hypothesis 1, the target localization gradients for the three groups were nearly parallel and the No-CI group’s gradient in particular showed no evidence of flattening relative to the hearing group’s gradient.

### Localization RT Group Comparisons

The RT data for the localization task were submitted to the same full set of analyses described above for the localization task accuracy data, including unadjusted analyses, analyses adjusted for demographic variables only, and analyses further adjusted for central identification accuracy and executive function. There were no significant differences or trends among groups to support the existence of a peripheral advantage or a flattened gradient of attention in speed of processing for the deaf participants under conditions of divided attention.

### Correlations between Time-with-Implant and Task Performance

The correlation between time-with-implant (chronological age minus age of implantation) and central identification task accuracy, consistent with Smith et al. (1998) for children 4–7 years old, was small and not significant, *r* = 0.04, *t*(9) = 0.11, *p* = 0.9114, even after adjusting for demographic variables, adjusted *r* = 0.07, *F*(1,5) = 0.08, *p* = 0.7855. Correlations between time-with-implant and accuracy at parafoveal and peripheral locations, or averaged over both locations did not reach significance, parafoveal localization accuracy: unadjusted *r* = 0.51, *t*(9) = 1.8, *p* = 0.1082, adjusted *r* = 0.46, *F*(1,5) = 1.7, *p* = 0.2453; peripheral localization accuracy: unadjusted *r* = 0.28, *t*(9) = 0.9, *p* = 0.4130, adjusted *r* = 0.52, *F*(1,5) = 2.3, *p* = 0.1892; overall localization accuracy, unadjusted *r* = 0.29, *t*(9) = 0.91, *p* = 0.3857, adjusted *r* = 0.55, *F*(1,5) = 2.5, *p* = 0.1730. We considered the possibility that individual differences in general factors (e.g., overall perceptual-motor skills) might commonly affect performance across the two tasks, which might create substantial error variance that masked subtler partial correlations between time-with-implant and performance on specific tasks. In fact, central identification accuracy was highly correlated with parafoveal and peripheral localization accuracy within the CI group (*r* = 0.74 and 0.75, respectively), suggesting the presence of a substantial common source of error variance. Therefore, we included central identification accuracy and localization accuracy as covariates to control their common variance in a regression analysis to predict time-with-implant, along with the demographic covariates. Because parafoveal and peripheral accuracy were highly correlated (0.84), they were averaged together to avoid multicollinearity problems and to act as an estimate of overall localization task accuracy. After controlling this joint variance, overall localization accuracy was positively correlated with time-with-implant, adjusted *r* = 0.60, *F*(1,4) = 9.8, *p* = 0.0354. Higher localization accuracy was associated with longer time-with-implant over the range of 4 to 18 years. In addition, with localization task performance controlled, there was a trend for an independent negative correlation between central task performance and time-with implant, adjusted *r* = -0.44, *F*(1,4) = 5.3, *p* = 0.0828. Lower central task accuracy tended to be associated with longer time-with-implant over the range of 4–18 years. Parallel analyses between time-with-implant and RT produced correlations that were all small and did not approach significance.

### Correlations of Performance among Stimulus Locations within Groups

The dissociation of the No-CI group’s central identification deficit from their localization performance in the group analyses raises the possibility that auditory deprivation is associated with a decoupling of performance across tasks or visual field locations within individuals. This possibility led us to examine the intercorrelations among central task performance and performance at the parafoveal and peripheral eccentricities across participants within each group for evidence of such decoupling. **Table 2** confirms this possibility. Controlling for demographic variables, accuracy at all eccentricities was weakly to strongly correlated within the hearing and CI groups, but was not correlated within the No-CI group. Similarly, localization RTs at the parafoveal and peripheral eccentricities were strongly correlated with each-other, with nearly identical values for the hearing and CI groups. The parafoveal versus peripheral RT correlation for the No-CI group was significantly smaller than the same correlation for the other two groups combined (Fisher test, *p* < 0.05).

**Table 2:** Semipartial correlations of accuracy scores among central, parafoveal, and peripheral sites and of RT scores between parafoveal and peripheral sites within groups adjusted for age, gender, race/ethnicity, and childhood SES.

	Hearing		Deaf CI		Deaf No CI	
Accuracy	Parafoveal	Peripheral	Parafoveal	Peripheral	Parafoveal	Peripheral
Central	0.38^!^	0.56^∗^	0.60^∗^	0.58^∗^	0.02	0.14
Parafoveal		0.43^∗^		0.82^∗^		0.20

**Response time**	**Peripheral**		**Peripheral**		**Peripheral**	

Parafoveal	0.84^∗^		0.83^∗^		0.46^∗^	

*^∗^*p* = 0.05 or better; ^*!*^*p* < 0.1.*

## Discussion

This study used a novel divided attention paradigm requiring central object identification and concurrent spatial localization at parafoveal and peripheral locations. Our goal was to test the proposal that a general graded trade-off of attentional resources from central to peripheral locations across the visual field, due to auditory deprivation, can adequately explain the spatial distribution of deaf-hearing performance differences on attention-dependent tasks that has been reported in the literature. We used groups with different levels of auditory experience to test the prediction from this proposal that concurrent central object identification deficits and related peripheral spatial localization advantages would appear for deaf participants without, but not with, CI, compared with hearing participants, and that these effects would be linked to a flattening of the spatial gradient of attention.

Our results showed that deaf young adults without CIs, but not with CIs, displayed a significant deficit in central identification accuracy compared with hearing young adults. This central deficit was reliable in unadjusted analyses and in analyses with several common demographic variables controlled, as well as analyses with localization task performance and executive function further equated across groups. This result is consistent with the finding of central attentional deficits in several previous CPT studies of children and adults (Quittner et al., 1994; Mitchell and Quittner, 1996; Smith et al., 1998; Parasnis et al., 2003; Horn et al., 2005), in Proksch and Bavelier’s (2002) study using a central flanker interference task, and in previous divided attention studies involving a central identification task concurrent with a motion or orientation detection task (Bosworth et al., 2013) or a peripheral localization task (Dye, 2016).

Contrary to predictions, the three groups did not differ in peripheral localization accuracy or RT performance. Furthermore, neither deaf group’s localization gradient was significantly different from the hearing group gradient in unadjusted analyses, nor in analyses adjusted for demographic factors nor further adjusted for overall group central identification accuracy and executive function. These results agree with those of previous divided visual attention studies, which showed a central identification deficit but failed to show a peripheral advantage for deaf participants for attention-dependent motion processing (Bosworth et al., 2013) or target localization amidst distractors (Dye, 2016). The fact that statistically equating groups on their central identification performance did not cause an overall localization performance advantage or a flattening of the localization performance gradient to emerge for the No-CI deaf group implies that this group did not trade-off central attentional resources for enhanced attentional resources in the localization task generally or at peripheral locations specifically. Thus, central identification task performance and localization performance across visual space were functionally and statistically dissociated in our study, suggesting independent attentional regulation of ventral and dorsal stream tasks. In support of this interpretation, Bosworth et al. (2013) used a different pair of ventral and dorsal stream tasks (RSVP shape identification and coherent motion detection, respectively) and also presented evidence that the central object identification group deficit was independent of equivalent group performance on motion detection in both central and peripheral regions. However, it is worth noting that Bosworth et al. (2013) also did not find a central deficit on their concurrent ventral stream orientation task, suggesting that the central deficit might not be general over all ventral stream tasks. Generally, our results combine with previous divided attention studies to suggest that central object identification deficits are independent of peripheral task performance.

It is unlikely that the central object identification deficit can be explained by general sensory or perceptual skill deficits in our deaf participants. Dye (2016) reported that performance on his central identification task in the absence of an attentionally demanding concurrent peripheral task did not result in a central deficit in deaf participants, indicating that the central deficit found in his UFOV task was not associated with an overall deficit in sensory or perceptual skills. Although we did not test central vehicle identification performance in the absence of our localization task, the facts that all three groups displayed nearly identical performance on the localization task at the parafoveal location on the immediate edge of the central vehicle stimuli in both unadjusted and adjusted analyses, and that the deaf group with CI did not show a central deficit in this or previous studies, converge with Dye’s (2016) earlier finding to suggest that the central object identification deficit we observed cannot be easily attributed to unknown deafness-related differences in overall sensory or perceptual skills.

It is possible that population differences in specific attention demanding cognitive processes involved in identification tasks could account for the central deficit. For example, working memory demands have been a component of the central task in most CPT studies (but not all – see Parasnis et al., 2003) and some divided attention studies, and it has been proposed that deaf and hearing people have different processing biases in working memory depending on auditory deprivation (Hall and Bavelier, 2010). Similarly, other work suggests that deaf people exhibit deficits on various executive function measures including the BRIEF (Hintermair, 2013). However, our analyses controlling for BRIEF-A working memory and GEC scores tend to rule out group differences in working memory or other executive functions as a specific factor determining the central deficit in our participants. Generally, the selective appearance of a central object identification deficit in prelingually deaf college adults without but not with CI, no history of ADHD diagnosis or neurological disorders, normal or corrected vision, with common demographic characteristics and executive function skills controlled, and with equivalent performance on a competing dorsal stream localization task, suggests a specific deficit in central attention-dependent processes.

Another important consideration is that like most previous studies, we were not able to control for factors such as specific etiologies of deafness or early language delay. Dye and Hauser (2014) recently showed that 6–13 years old deaf children born to deaf parents who acquired ASL from birth did not show a central deficit on the Gordon CPT. These results suggest that language delay and/or unknown neurological consequences of non-genetic causes of deafness may account for the central deficits shown in previous CPT studies of sustained attention. Since these factors may have been present in some of our deaf participants, it remains possible that they represent the mechanism underlying the central deficit we observed in the No-CI group. However, against this interpretation is the fact that the No-CI and CI groups had statistically equivalent distributions of ages of onset of deafness, best language, and parental hearing status, with the numerical balance tipped in favor of more No-CI participants having deaf parents.

It is important to consider the possibility that a general altered gradient of attention could still explain a central deficit without a concurrent peripheral advantage. Although Proksch and Bavelier (2002), reported that deaf participants showed weaker interference by central distractors and hearing participants showed weaker interference by peripheral distractors, these effects were not completely linked in their study, but tended to emerge separately as a function of target search load. Proksch and Bavelier (2002) used target search sets ranging from 1 to 6 stimuli. At the smallest and largest search loads, deaf and hearing participants showed equivalent distraction effects. The weaker central distraction effect for deaf compared with hearing participants first appeared at a search load of 2, but the weaker peripheral distraction effect for hearing participants did not occur until a search load of 4. Proksch and Bavelier (2002) argued that, assuming a more limited allocation of attentional resources to central vision, deaf participants depleted their central attentional resources at a lower load than hearing participants, but both groups had sufficient peripheral attentional resources to produce peripheral distractibility effects at that load. At a higher load, hearing participants depleted their more limited peripheral attentional resources before deaf participants did, selectively eliminating the distractibility effect for that group. However, if Proksch and Bavelier (2002) had measured distractor effects only at the smaller intermediate load, they would have seen a weaker central distractor effect for their deaf participants without a corresponding weaker peripheral distractor effect for their hearing participants, which would have appeared to suggest a central attentional deficit for deaf participants without a corresponding peripheral advantage.

One could argue therefore that, assuming attentional resources are redistributed from central to peripheral vision, the difficulty of our tasks was such that the No-CI deaf group’s available central attentional resources might have been insufficient to match the hearing groups central identification performance, but that both groups’ peripheral attentional resources might have been sufficient to maintain equivalent peripheral localization performance. Since we did not vary task difficulty, we might not have seen a peripheral advantage emerge for our No-CI group at a higher difficulty level. This argument might be consistent with data from studies that only sample performance at one central and one peripheral eccentricity, separated by significant distance. However, standard attentional gradient models (Greenwood and Parasuraman, 1999; Bush, 2012) predict that a general flattened attention gradient should still smoothly allocate very similar levels of attentional resources at closely adjacent eccentricities everywhere along the gradient, for example at the closely adjacent central and parafoveal locations used in our study. Indeed, the hearing group’s central identification and parafoveal localization accuracies were nearly identical, suggesting that the two tasks are equivalent in difficulty under conditions of similar allocation of attentional resources along the gradient at those closely adjacent locations. Given the dramatic deficit in central vehicle identification performance shown by the No-CI deaf group (approximately 10%), their sudden jump of approximately 11% to an equivalent performance level as the hearing group at the parafoveal eccentricity on the very edge of the central vehicle stimuli is not consistent with a flattened continuous gradient of attentional resources across those adjacent locations. Rather, their significant discontinuous local jump in performance, the lack of evidence for a flatter attentional gradient from parafoveal to peripheral locations, and the statistical dissociation of group differences across the two tasks near the center of vision suggest that the central identification deficit was specific to the processing demands of the identification task. These results therefore favor the proposal that central deficits and peripheral advantages may be independently determined by distinct mechanisms, an alternative interpretation suggested by Proksch and Bavelier (2002).

Dye (2016) attempted to explain the presence of a central deficit without a peripheral advantage in his data by proposing that the fine-scale resolution required for central object discrimination in his task may have biased participants to prioritize central processing at the expense of enhanced peripheral attention. Accordingly, deaf participants may have still drawn some resources from central vision to maintain equivalent peripheral performance but not enough to produce superior peripheral performance to hearing participants. This explanation appears to preserve a form of the altered gradient proposal since central resources are still redistributed toward the periphery. However, there are two problems with this explanation. The first is theoretical. It has been argued that deaf/hearing differences in performance on visual tasks may be better characterized as a generalized attentional difference rather than as resulting from deficient visual cognition (Bavelier et al., 2006). This position seemingly implies that deaf and hearing individuals have the same total available attentional resources and that the redistribution of visual attentional resources in deaf individuals is an adaptive zero-sum game since it results in local advantages and deficiencies but not a general deficiency. That is, the original altered gradient of attention hypothesis is a mechanism proposed to explain deaf-hearing differences in performance on visual tasks while preserving the proposal of total non-deficiency. However, given the presence of a reliable central deficit, Dye’s (2016) explanation seems to imply that the total available attentional resources for allocation between central and peripheral eccentricities in tasks such as his may be smaller in deaf adults than in hearing adults, since the extra resources taken from central vision are hypothetically only sufficient to elevate peripheral performance to equivalent hearing population levels. This position therefore undermines the motivation for the original altered gradient of attention hypothesis. The second problem is that our present results did not reveal an altered gradient of attention in the localization task, or a statistical coupling of the central deficit to peripheral group performance, even allowing for a possible overall population deficit in total attentional resources.

It has also been proposed that in situations where deaf people must allocate attention across the visual field, they may shift the peak focus of their attention to peripheral locations (Dye and Bavelier, 2013). However, following standard models of the spread of the gradient of attention (LaBerge and Brown, 1989; Staugaard et al., 2016), this proposal still amounts to the claim that performance at most eccentric points in the visual field should not be equivalent in deaf and hearing people, and therefore, the slopes of the gradients of attention measured between identical eccentricities should generally not be the same, even if attention is focused at different points in space for the two groups. Therefore, our localization gradient data are inconsistent with a simple refocusing of the locus of attention to an eccentric location due to auditory deprivation.

It is also possible that we simply did not test for deaf-hearing differences far enough out in the visual field. However, Dye (2016) tested at 20° and did not find a peripheral advantage and peripheral advantages have been shown in other studies as close as 2–3° (Parasnis and Samar, 1985; Pavani and Bottari, 2012). Nevertheless, there is animal evidence that the gradients of attention for deaf and hearing groups can run parallel for a considerable distance out from central fixation and then diverge with a shallower slope for deaf individuals. Lomber et al. (2010) reported parallel and equivalent performance of deaf and hearing cats in a localization task over the range of 0–45°. However, farther from central vision (45–90°), hearing cats showed a steep graded drop in localization performance while deaf cats showed a shallower, more even decrease, resulting in an increasingly larger perceptual advantage with increasingly extreme eccentricity. Since we tested over only part of the visual field (up to 13.3°), we cannot be certain that our deaf No-CI participants would not show a peripheral advantage in the localization task farther out in the visual field. Indeed, as in previous studies, our No-CI group must presumably still have monitored their full visual field beyond the computer screen for unexpected environmental events while they were simultaneously monitoring the demanding events on the spatially limited screen in our experiment. It could be argued that the participants in our study, and in previous studies that have not shown peripheral advantages, broadly partitioned resources taken from central vision to cover both the eccentric regions of the computer display and the rest of their visual environment, and that the peripheral advantage would have shown up if we had tested farther out in the field. However, given the discontinuous jump in performance for the No-CI group specifically between the central identification task and the localization task at the 1.4° parafoveal location, an undetected and more remote peripheral advantage would still not be easily explained by a simple broadening of a general gradient of attention. Furthermore, Lomber et al.’s (2010) deaf cats showed no central deficit at 0°, suggesting that their peripheral advantage did not stem from a specific smooth but broad tradeoff of fixed attentional resources across the visual field. Rather, using localized cortical deactivation, Lomber et al. (2010) showed that the performance advantage was caused by cross-modal reorganization of a specific region of auditory cortex. Superior motion detection in the same animals was shown to be caused by specific cross-modal reorganization of a different region of auditory cortex. Deactivation of the motion detection cortex did not alter the cats’ localization performance anywhere in the visual field, indicating that their peripheral advantage in the localization task was not due to an enhancement of general attentional resources at peripheral locations but was task specific. Thus, their results suggest that the mechanism underlying deaf-hearing differences in visual attentional performance gradients is not a general redistribution of attentional resources across the visual field that affects all attention-demanding tasks, but rather a functionally specific cross-modal reorganization of multiple neurobiologically distinct systems that may independently regulate performance at different points in the visual field for specific purposes.

Lomber et al.’s (2010) results are consistent with growing evidence that functionally specific plastic changes occur at different loci within sensory and polymodal cortical systems in response to sensory deprivation (Bavelier and Neville, 2002), and that functionally adaptive attention-dependent changes occur within both dorsal and ventral stream systems (Weisberg et al., 2012), giving rise to specific patterns of deaf-hearing differences under different experimental conditions. This specificity appears to be a natural outgrowth of the highly differentiated organization of the attentional system and its interaction with cross-modal processes. The distribution of attentional resources within and across modalities and tasks is driven by competition modulated in part by specific task demands and stimulus features (Rapp and Hendel, 2003). Normally, cross-modal integration continuously interacts with the attentional system to increase the amount of convergent information available to the visual system for detecting and interpreting complex environmental events (Talsma et al., 2010). For example, many studies have shown that auditory stimulation substantially enhances basic visual perception, including regulating brightness perception, improving the speed and accuracy of target detection, increasing temporal resolution, facilitating motion perception, and modulating attentional processes (Shams and Kim, 2010). Furthermore, spreading responsibility for processing multiple events across modalities, especially those involving the same stimulus features, results in less competition for total limited attentional resources than performing the same tasks within that modality (Rapp and Hendel, 2003). Thus, cross-modal integration essentially extends the visual system’s degrees of freedom for multitask processing while reducing multitask competition.

By the same token, the loss of auditory cross-modal integration may increase competition between tasks when they must be accomplished within the visual modality instead of cross-modally. Evidence suggests that under these circumstances of heightened intra-modal competition, the visual system adaptively adds capacity by mechanisms such as recruitment of multisensory or primary sensory cortex (Bavelier et al., 2006; Hirst et al., 2012; Karns et al., 2012). Added capacity heightens the internal differentiation of the visual attention system, essentially transforming cross-modal degrees of freedom from the auditory system into intramodal degrees of freedom in the visual system. This increased organizational complexity would permit more flexible, fine-grained coordination of attentional resources within the visual system across tasks and stimulus feature representations as well as at different eccentricities for different tasks, leading to a different attentional architecture to achieve the same goal-directed adaptive results that would ordinarily be accomplished by cross-modal integration mechanisms. In particular, Lomber et al.’s (2010) demonstration in deaf cats of a far, but not near, visual field flattening of the gradient for visual localization due to specific recruited mechanisms in auditory cortex provides evidence for an increased capacity to flexibly regulate attentional resources across distant local regions of the visual field. In general, current evidence supports the view that visual system mechanisms become increasingly differentiated in response to specific competitive behavioral and cognitive pressures associated with auditory deprivation (Bavelier and Neville, 2002; Lomber et al., 2010; Weisberg et al., 2012). These considerations generally suggest that deaf-hearing differences in the distribution of spatial attention are likely to be task and goal dependent and precisely regulated by multiple distinct neural subsystems rather than driven by a redistribution of general attentional resources that applies across tasks.

The finding that time-with-implant did not have a simple correlation with performance in either the central identification task or the localization task at any eccentricity for our adult participants extends Smith et al.’s (1998) previous correlational findings with children to adults and to an eccentric localization task. Smith et al. (1998) replicated Quittner et al.’s (1994) finding that CIs improved CPT performance in children relatively rapidly (within about 18 months), but also reported that, unexpectedly, time-with-implant did not correlate with CPT performance over a range of times-with-implant as brief as 6 months to 2 years. They argued that the lack of correlation is consistent with the claim that the relevant effects of the implant occur rapidly. Our CI participants had their implants for a minimum of 4 years and a maximum of 21 years. Hence our findings are also consistent with the assumption that adults with CI may have experienced a rapid reversal of central attentional deficits following implantation.

Given that restored attentional skill may occur less than 2 years after implantation, the lack of simple correlations between time-with-implant and central and peripheral task performance provides no evidence *per se* for or against the altered gradient proposal. However, our finding of independent semi-partial positive correlations raises the possibility that some aspects of cross-modal plasticity related to restored auditory input may operate on a longer maturational time scale. The general gradient of attention proposal does not appear to predict these correlations. If anything, it would predict that with increased time-with-implant, dorsal stream peripheral accuracy enhancements would be reduced and ventral stream central accuracy deficits would be improved, not the other way around. On the contrary, the independent semi-partial correlations we observed suggest that some aspects of dorsal and ventral stream processes may be differentially affected by restored auditory input over long periods during childhood. It appears that performance on dorsal stream tasks may partially improve slowly over several years of CI use. Somewhat paradoxically, central ventral stream performance may independently partially decrease slowly with long-term CI use. This is an issue for future research. Note, however, time-with-implant strongly reflects age of implantation during childhood in our sample (*r* = 0.93) which ranged from 1.5 to 17 years old. Therefore, these effects may reflect separate interactions between the timing of restored auditory input and developmental stages or critical periods of specific ventral and dorsal stream processes. This interpretation is consistent with studies showing that plastic changes associated with sensory deprivation are shaped by specific experience interacting with the timing of critical periods and other maturational factors (Bavelier and Neville, 2002).

The correlations in **Table 2** suggest that auditory deprivation may decouple, and restored auditory input may recouple, overall performance across tasks and visual field locations. The No-Ci group had uniformly low, non-significant accuracy intercorrelations among tasks and eccentricities and a significantly lower correlation between parafoveal and peripheral RT than the other groups. Absent or reduced correlations for the No-CI group are not explained by less reliable general performance than the other groups. If that were true, we would likely expect overall worse performance and increased variability in that group compared with the other groups. While the No-CI group displayed worse central object identification performance, the three groups had closely equivalent RTs and high accuracy scores at both eccentricities in the localization task. In addition, the No-CI group’s variance was at or numerically below that of the CI group on both tasks (Accuracy: *p*’s = 0.8546 to 0.9884; RT: *p*’s = 0.9586 to 0.9612). Another explanation, which would be consistent with the altered gradient of attention model, is that individual differences in the availability of residual auditory input might have induced within-group variability in the No-CI participants’ gradient-of-attention slopes, whereas the restoration of auditory input might have reduced such within-group variability in CI participants’ gradients. However, the No-CI and CI group variances of the parafoveal-peripheral performance difference scores were numerically close and did not differ significantly (accuracy: *p* = 0.3975; RT: *p* = 0.4388), indicating no evidence that the No-CI group’s absent or low correlations reflect individual variation in attention gradient slopes. We propose instead that the selective decorrelation of performance across tasks and sites is consistent with the claim that cross-modal plasticity following auditory deprivation increases the visual system’s capacity to independently regulate attention on a finer scale across visual space in order to maintain optimal task performance under competing task conditions.

It is important to remember that, although we controlled several background factors that might impact group differences, it was not possible to match participants across groups on all potentially relevant personal characteristics and developmental factors. The heterogeneous nature of the deaf community, including the potential developmental impact of different causes of deafness, audiometric functional complexity, and educational, family, social, and linguistic history present significant challenges to research designed to experimentally characterize the behavioral and neurobiological consequences of auditory deprivation. Understanding the impact of these factors on attentional performance in the larger deaf community will require increased emphasis on experimental controls and sampling practices in future studies.

## Conclusion

Our results converge with previous divided visual attention studies that failed to demonstrate a trade-off of attentional resources from central to peripheral visual field locations in deaf people who grow up without access to functional auditory input. In the context of the larger literature that documents inconsistent reports of deaf-hearing advantages, deficits, and equivalencies, our results suggest that a general graded trade-off of attentional resources across the visual field cannot adequately explain the complex task-dependent spatial distribution of deaf-hearing performance differences. Rather, our results are consistent with growing neurobiological evidence that the spatial distribution of attention-mediated performance in deaf people is determined by sophisticated cross-modal plasticity mechanisms that recruit specific sensory and polymodal cortex, increasing intra-modal visual processing capacity and attentional control to achieve specific compensatory behavioral goals.

## Author Contributions

LB and VS conceived and designed the research and analyzed data. LB recruited participants and collected the data. VS primarily wrote the paper with critical editing contributions for intellectual content from LB.

## Conflict of Interest Statement

The authors declare that the research was conducted in the absence of any commercial or financial relationships that could be construed as a potential conflict of interest. The reviewer RPG and handling Editor declared their shared affiliation, and the handling Editor states that the process nevertheless met the standards of a fair and objective review.
